# The center of pressure position in combination with ankle dorsiflexion and trunk flexion is useful in predicting the contribution of the knee extensor moment during double-leg squatting

**DOI:** 10.1186/s13102-022-00523-0

**Published:** 2022-07-14

**Authors:** Tomoya Ishida, Mina Samukawa, Satoshi Kasahara, Harukazu Tohyama

**Affiliations:** Faculty of Health Sciences, Hokkaido University, North 12, West 5, Kitaku, Sapporo, 060-0812 Japan

**Keywords:** Squat, Exercise, Compensation, Biomechanics, Asymmetry

## Abstract

**Background:**

Squatting exercises are commonly used in rehabilitation for knee joint disorders; in these exercises, control of knee extensor moment is important to enhance training effects and to avoid adverse effects. Ankle dorsiflexion and trunk flexion are widely used to reduce knee extensor moments during squatting, but the increased load on the low back is a concern. The purpose of this study was to determine whether the anterior–posterior (AP) center-of-pressure (COP) position and the AP-COP position in combination with ankle dorsiflexion and trunk flexion angles can predict the contribution of the knee extensor moment during double-leg squatting.

**Methods:**

Twenty-eight healthy individuals (14 female and 14 male participants, age 22.8 ± 1.3 years) performed three sets of five consecutive double-leg squats. Kinematics and kinetics were analyzed using a three-dimensional motion analysis system with force plates. Univariate and multivariate regression analyses were performed to predict the contribution of the knee extensor moment (% total support moment) from AP-COP position, ankle dorsiflexion, and trunk flexion.

**Results:**

The AP-COP position was a significant predictor of the knee extensor moment contribution (*R*^2^ = 0.168, *P* = 0.030). Multivariate analysis showed that the ankle dorsiflexion angle (*ΔR*^2^ = 0.561, *β* = 0.842) and AP-COP position (*ΔR*^2^ = 0.296, *β* =  − 0.499) predicted the knee extensor moment contribution (model *R*^2^ = 0.857, *P* < 0.001). Additionally, the combination of trunk flexion (*ΔR*^2^ = 0.429, *β* =  − 0.613) and AP-COP position (*ΔR*^2^ = 0.109, *β* =  − 0.332) predicted the knee extensor moment contribution (model *R*^2^ = 0.538, *P* < 0.001). The limb symmetry index of the knee extensor moment was significantly associated with that of the AP-COP position (*R*^2^ = 0.493, *P* < 0.001) but not with that of the ankle dorsiflexion angle (*P* = 0.057).

**Conclusions:**

The AP-COP position can predict the contribution of the knee extensor moment and improve the prediction when combined with ankle dorsiflexion and trunk flexion. The present findings suggest that intervention focusing on the AP-COP position in combination with ankle dorsiflexion or trunk flexion would be useful to coordinate the contribution of the knee extensor moment during double-leg squatting.

## Introduction

Squatting exercise is frequently and widely used to train the lower limb muscles in rehabilitation and conditioning [1]. The descent and ascent of the center of body mass during squatting is achieved by hip, knee, and ankle extensor moments [2]. Therefore, the contribution ratio of the hip, knee and ankle extensor moments during squatting can differ among individuals [2]. To enhance the effect of exercise, each hip, knee and ankle joint contribution should be adjusted to the target of exercise [1]. Moreover, coordination of each joint contribution during squatting should be considered to prevent adverse effects in rehabilitation, especially for knee joint disorders [2–5]. For example, after anterior cruciate ligament reconstruction, patients showed a smaller contribution of the knee extensor moment relative to the hip and ankle extensor moments during double-leg squatting [2–5]. At 13 months postoperatively, the patients demonstrated a knee-to-total support moment ratio of 36% in the involved limb and 41% in the uninvolved limb, compared with 44% for healthy controls [2]. Although such a smaller knee contribution has an advantage in the acute phase to avoid interfering with graft healing, the increase in contribution of knee extensor moment has the advantage of increasing quadriceps strength for people returning to sports [2–5]. For patellar tendinopathy, load management of the tendon is also important for the treatment according to symptoms [6]. Patients with patellofemoral pain syndrome should also coordinate their knee extensor moments to decrease patellofemoral joint stress [7]. Clinically, it is important to treat the knee extensor moment carefully in knee joint disorders.

The effects of ankle dorsiflexion and the resulting anterior knee position on the knee extensor moment during double-leg squatting, as well as the effects of trunk flexion, have been well studied [7–11]. Reduced ankle dorsiflexion and increased trunk flexion decrease the knee extensor moment while increasing the hip extensor moment during double-leg squatting [7–11]. However, increased trunk flexion is concerned about the increase in the load on the low back [10, 12]. Reduced ankle dorsiflexion is also associated with an increase in hip flexion angle, hip extensor moment and lumbar lordosis [7, 10, 12], which is related to the increase in the load on the hip joint and low back [10, 12]. Therefore, strict restriction of ankle dorsiflexion alone is also not recommended [12]. It is important to adjust the knee extensor moment by considering other factors in addition to ankle dorsiflexion and trunk flexion to avoid increasing the load on the low back.

A previous study showed that a more anterior center-of-pressure (COP) position was associated with a smaller contribution of the knee extensor moment relative to the hip and ankle extensor moments during double-leg squatting after anterior cruciate ligament reconstruction [5]. The anterior-posterior (AP)-COP position is thought to affect lower-limb joint moments during squatting by changing the distance between the ground reaction force vector and the lower-limb joints [5]. Therefore, there is a possibility that a more anterior COP position is associated with a smaller contribution of the knee extensor moment during double-leg squatting. However, it is unclear whether the AP-COP position alone and in combination with ankle dorsiflexion and trunk flexion angles can predict the contribution of the knee extensor moment during double-leg squatting. If the AP-COP position can predict the contribution of the knee extensor moment independently of ankle dorsiflexion and trunk flexion angles in multivariate analysis, it may be possible to adjust the knee extensor moment without strictly limiting ankle dorsiflexion or increasing trunk flexion.

In addition, significant interlimb asymmetry in the knee extensor moments was reported even for healthy individuals [13]. The limb symmetry index (LSI) of the knee extensor moments was significantly associated with the LSI of the AP-COP position during double-leg squatting after anterior cruciate ligament reconstruction [5]. However, the mean LSI of patients was 67% [5], which seems to be smaller than the LSI of healthy people (87–90%) [13]. Although no clear evidence exists, there is concern that biomechanical asymmetry may be associated with musculoskeletal injuries [13, 14]. Therefore, it is important to examine whether interlimb asymmetry in the AP-COP position can predict asymmetry in the knee extensor moments in healthy individuals.

The purpose of the present study was to determine whether the AP-COP position alone and in combination with ankle dorsiflexion and trunk flexion angles can predict the contribution of the knee extensor moment during double-leg squatting. In addition, the relationships between the AP-COP position and ankle dorsiflexion and trunk flexion were examined. The hypotheses were that adding the AP-COP position to ankle dorsiflexion and trunk flexion would improve prediction of the knee extensor moment and that the LSI of ankle dorsiflexion and the AP-COP position would predict the LSI of the knee extensor moment.

## Methods

### Participants

Based on previous studies [5, 11, 13], a coefficient greater than 0.3 in the multiple regression model was anticipated to predict lower-limb extensor moments. To achieve a significance level (*α*) and statistical power (1 − *β*) of 0.05 and 0.8, respectively, 27 participants were needed. Therefore, the present study enrolled 28 participants (14 female and 14 male participants, age 22.8 ± 1.3 years, height 167.8 ± 8.0 cm, and body mass 58.8 ± 10.1 kg). Participants were excluded from this study if they reported pain during double-leg squatting, had any history of musculoskeletal injury within the prior 6 months, or had surgery of the lower extremities or the trunk. Written informed consent was obtained from each participant before participation. This study was approved by the Institutional Review Board of Faculty of Health Sciences, Hokkaido University (approval number: 19-72).

### Procedures and data collection

Participants warmed up using a stationary bicycle ergometer at a self-selected pace for five minutes. Then, retroreflective markers were placed on the iliac crest, anterior and posterior superior iliac spines (ASISs and PSISs, respectively), lateral thigh, medial and lateral femoral epicondyle, lateral shank, medial and lateral malleoli, second metatarsal head and base, fifth metatarsal head and heel. Following a static standing trial, participants performed three sets of five consecutive double-leg squats. They squatted down until their thighs were parallel to the floor and then stood upright [15]. If their heels came off the floor, they were instructed to squat as deeply as possible without their heels coming off the floor. No specific instructions were given regarding trunk flexion. The descent and ascent phases were set to 2 s each using a metronome. Participants were asked to place one foot on an individual force plate with their feet shoulder width apart and to cross their arms over their chests. Two to three minutes of rest was allowed between each trial.

A motion capture system (Cortex version 5.0.1, Motion Analysis Corporation, Santa Rosa, CA, USA) with seven high-speed digital cameras (Hawk cameras, Motion Analysis Corporation) and two synchronized force plates (Type 9286, Kistler AG, Winterthur, Switzerland) were used for data recording. The sampling rates were set to 200 Hz for the marker trajectory data and 1000 Hz for the force plate data.

### Data processing and reduction

Kinematic and kinetic analyses were performed using Visual3D (version 6, C-Motion, Inc., Germantown, MD, USA). Marker trajectories and force plate data were low-pass filtered using a fourth-order, zero-lag Butterworth filter with a 12 Hz cutoff frequency [4, 5]. The trajectory gaps of ASIS markers during the squatting position were filled using iliac crest and PSIS markers [16]. Trunk flexion and lower-limb joint angles were calculated using a joint coordinate system with the Cardan sequence. The trunk flexion angle was determined relative to the laboratory coordinate system. The knee extensor moment was calculated using an inverse dynamics approach, and the segment inertial properties were set according to a previous report [17]. The present study examined the knee extensor moment contribution because the absolute knee extensor moment can also be affected by the squatting speed and interlimb difference in weight bearing [1, 13, 18]. To determine the knee extensor moment contribution, the knee extensor moment was normalized to the total support moment, which was the sum of the hip, knee and ankle extensor moments (% total support) [3]. The COP position of each foot was calculated for AP direction. The AP direction was adjusted by the vector from the heel marker to the 2nd metatarsal head marker. The AP-COP position was expressed as the percentage of the foot length (% foot length) from the heel marker (0%) to the second metatarsal head marker (100%).

The middle three of the five consecutive squats were analyzed [4]. The knee extensor moment, ankle dorsiflexion angle, trunk flexion angle and AP-COP position at peak knee flexion were used in the subsequent statistical analysis [19]. Interlimb asymmetry was assessed using the LSI, which was calculated as the percentage of the value of the dominant limb to that of the nondominant limb. The dominant leg was determined as the side preferable for kicking a ball. All variables were averaged across the three squats of the three trials.

### Statistical analysis

All data are presented as the mean and standard deviation (SD). Univariate regression analysis was conducted to confirm the linear relationship between the knee extensor moment contribution and the AP-COP position and ankle dorsiflexion and trunk flexion angles. In addition, the linear relationships between the AP-COP position, ankle dorsiflexion angle and trunk flexion angle were also examined. Then, multivariate regression analysis was performed to determine the predictive ability of AP-COP position independent of ankle dorsiflexion and trunk flexion angles. A regression model of ankle dorsiflexion with trunk flexion was also tested. Analysis was performed regarding the dominant side, nondominant side and LSI. A paired t test was also conducted to confirm differences in variables of interest between the dominant and nondominant sides. The statistical significance level was set at *P* < 0.05. These statistical analyses were performed using JMP Pro software (version 15, SAS Institute Inc., Cary, NC, USA).

## Results

**Fig. 1:**
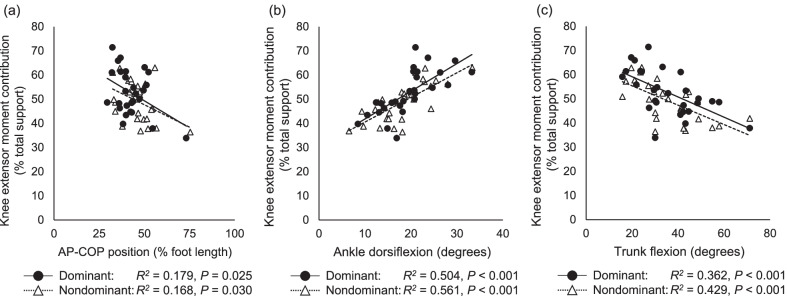
Relationship between the knee extensor moment contribution and the AP-COP position (**a**), ankle dorsiflexion angle (**b**) and trunk flexion angle (**c**). *AP-COP* anterior–posterior center of pressure

**Fig. 2:**
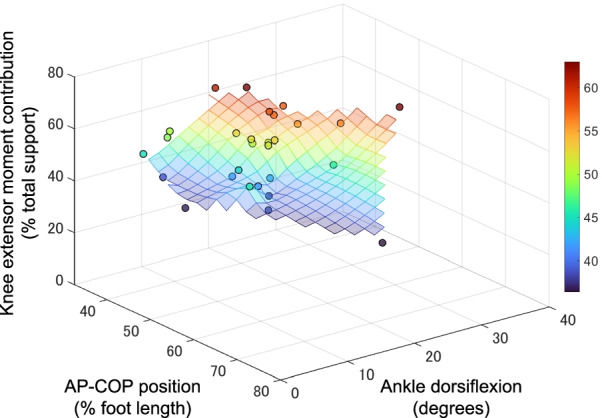
Color maps depict how the AP-COP position affects the knee extensor moment when combined with the ankle dorsiflexion angle. As shown in the multivariate regression analysis, the knee extensor moment tends to increase as the COP positions posteriorly (a smaller value of AP-COP indicates a more posterior position) along the axis of AP-COP position. Data for the nondominant side are shown. *AP-COP* anterior–posterior center of pressure

**Fig. 3:**
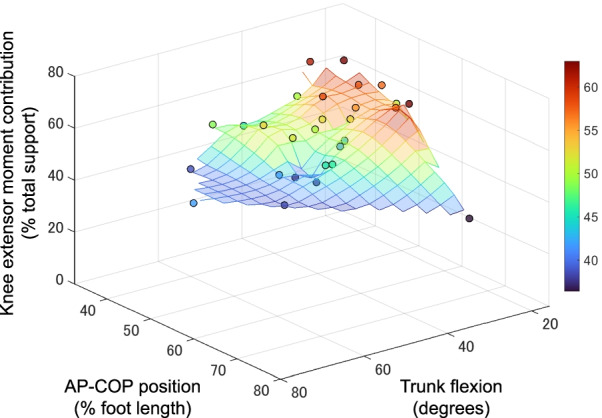
Color maps depict how the AP-COP position affects the knee extensor moment when combined with the trunk flexion angle. As shown in the multivariate regression analysis, the knee extensor moment tends to increase as the COP positions posteriorly (a smaller value of AP-COP indicates a more posterior position) along the axis of the AP-COP position. Data for the nondominant side are shown. *AP-COP* anterior–posterior center of pressure

Univariate analysis showed that the AP-COP position was significantly negatively associated with the knee extensor moment contribution (*P* < 0.05) (Fig. 1a). The ankle dorsiflexion and trunk flexion angles were also significant predictors of the knee extensor moment (*P* < 0.001) (Fig. 1b, c). Moreover, AP-COP position and ankle dorsiflexion angle independently predicted the contribution of the knee extensor moment in the multivariate analysis (dominant: model *R*^2^ = 0.752, *P* < 0.001; nondominant: model *R*^2^ = 0.857, *P* < 0.001) (Table 1). The knee extensor moment was positively associated with the ankle dorsiflexion angle but negatively associated with the AP-COP position (Fig. 2). The AP-COP position and trunk flexion angle also predicted the knee extensor moment contribution (dominant: model *R*^2^ = 0.533, *P* < 0.001; nondominant: model *R*^2^ = 0.538, *P* < 0.001) (Table 1). The knee extensor moment was negatively associated with the trunk flexion angle and AP-COP position (Fig. 3). The ankle dorsiflexion angle combined with the trunk flexion angle also significantly predicted the knee extensor moment (dominant: model *R*^2^ = 0.558, *P* < 0.001; nondominant: model *R*^2^ = 0.600, *P* < 0.001) (Table 1). However, the trunk flexion angle was not a significant predictor in the model of the dominant side. There was no significant association between the AP-COP position and the ankle dorsiflexion or trunk flexion angles (*P* = 0.393–0.939).

**Table 1:** Multivariate regression models to predict the knee extensor moment contribution

	Dominant side				Nondominant side			
	*ΔR*^2^	*B*	*β*	*P* value	*ΔR*^2^	*B*	*β*	*P* value
Model 1								
Ankle dorsiflexion, degrees	0.504	1.228	0.761	**< 0.001**	0.561	1.145	0.842	**< 0.001**
AP-COP, % foot length	0.248	− 0.534	− 0.500	**< 0.001**	0.296	− 0.499	− 0.551	**< 0.001**
Model 2								
Trunk flexion, degrees	0.362	− 0.414	− 0.595	**< 0.001**	0.429	− 0.375	− 0.613	**< 0.001**
AP-COP, % foot length	0.171	− 0.442	− 0.414	**0.006**	0.109	− 0.301	− 0.332	**0.023**
Model 3								
Ankle dorsiflexion, degrees	0.504	0.878	0.515	**0.002**	0.522	0.757	0.557	**< 0.001**
Trunk flexion, degree	0.064	− 0.211	− 0.330	0.066	0.073	− 0.203	− 0.332	**0.035**

Bold indicates significant predictors of the knee extensor moment contribution (% total support)

*ΔR*^2^ indicates the change in *R*^2^ for each step in the stepwise analysis

*AP-COP* anterior–posterior center of pressure

**Fig. 4:**
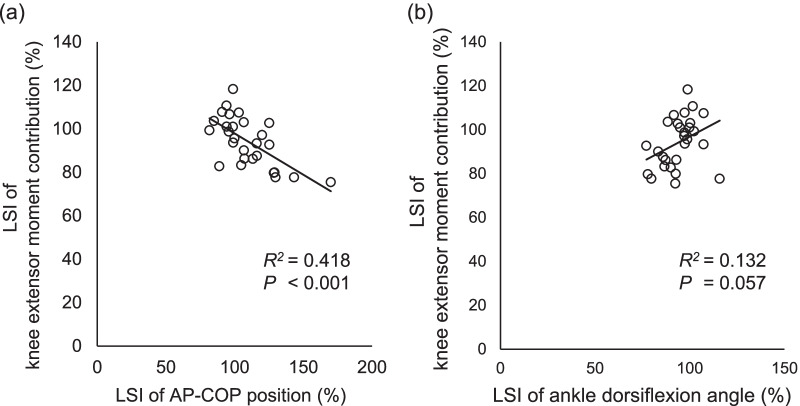
Relationship between the LSIs of the knee extensor moment and AP-COP position (**a**) and ankle dorsiflexion angle (**b**). *LSI* limb symmetry index, *AP-COP* anterior–posterior center of pressure

Significant interlimb differences were found for all interesting variables (Table 2). The LSI of knee extensor moment was significantly predicted by the LSI of the AP-COP position (*R*^2^ = 0.418, *P* < 0.001) but not by the LSI of the ankle dorsiflexion angle (*R*^2^ = 0.132, *P* = 0.057) (Fig. 4). Multivariate analysis showed that only the LSI of the AP-COP position was a significant predictor of the LSI of the knee extensor moment (*R*^2^ = 0.493, *P* < 0.001; AP-COP position: *P* < 0.001, *β* =  − 0.607; ankle dorsiflexion: *P* = 0.065, *β* = 0.278).

**Table 2:** Interlimb comparisons of variables of interest

	Dominant side	Nondominant side	LSI	95% CI	*P* value
Knee extensor moment, % total support	52.6 (9.2)	49.1 (8.1)	94.1 (11.4)	[1.1, 5.9]	**0.006**
Ankle dorsiflexion, degrees	19.4 (6.0)	18.4 (5.7)	94.1 (9.0)	[0.4, 1.6]	**0.003**
AP-COP, % foot length	42.7 (8.7)	46.2 (9.0)	109.6 (19.4)	[− 6.1, − 0.7]	**0.015**

Mean (SD)

Bold indicates a significant difference (*P* < 0.05)

*LSI* limb symmetry index

## Discussion and implications

The present study revealed that the AP-COP position significantly predicted the knee extensor moment contribution during double-leg squatting in univariate analysis and multivariate analysis when combined with the ankle dorsiflexion and trunk flexion angle. In addition, the LSI of the AP-COP position was found to be the only predictor of the LSI of the knee extensor moment contribution. These findings support the a priori hypothesis.

The AP-COP position was significantly negatively associated with the knee extensor moment contribution, while the ankle dorsiflexion angle was significantly positively associated with the knee extensor moment contribution; the trunk flexion angle was negatively associated. Furthermore, the addition of the AP-COP position to the ankle dorsiflexion and trunk flexion angle significantly improved the prediction of the knee extensor moment contribution. The ankle dorsiflexion angle explained approximately 50–56% of the variance in the knee extensor moment contribution, and the AP-COP position added 25–30% of the explanation. In the model combining the AP-COP position and trunk flexion, the trunk flexion angle explained approximately 36–43% of the variance in the knee extensor moment contribution, and the AP-COP position contributed another 11–17%. Moreover, the coefficient of determination of the model combining the AP-COP position and ankle dorsiflexion and trunk flexion angle was equal to or higher than that of the model combining the ankle dorsiflexion angle and trunk flexion angle, which has been widely used for the assessment of squatting [8, 11]. The present study found that the AP-COP position is useful in predicting knee extensor moment contribution independent of the ankle dorsiflexion and trunk flexion angles.

As shown in previous studies [8–11], the present results showed that a larger ankle dorsiflexion angle was associated with larger knee extensor moment contributions. On the other hand, a more anterior COP position was associated with a smaller knee extensor moment contribution. The ankle dorsiflexion angle and AP-COP position would influence the positional relationship between the knee joint and ground reaction force during squatting, predicting the knee extensor moment contribution. Although a previous study showed that double-leg squatting with restriction of ankle dorsiflexion demonstrated more posterior AP-COP position than double-leg squatting without restriction of ankle dorsiflexion [7], there was no significant association between the AP-COP position and the ankle dorsiflexion angle in the present study. This difference between the present and previous studies would be observed because the present study examined the squatting task in a participant’s own manner without restriction of ankle dorsiflexion. However, the present findings suggest that the AP-COP position is independent of the ankle dorsiflexion angle during the squatting task without restriction of ankle dorsiflexion. Therefore, these findings also support that the AP-COP position would be useful to predict the knee extensor moment contribution in combination with the ankle dorsiflexion angle.

Furthermore, the AP-COP position predicted the knee extensor moment independently of the trunk flexion angle in the multivariate model, and there was no significant association between the AP-COP position and trunk flexion angle. These findings suggest that the AP-COP position may be modified without changing the trunk flexion angle and may be useful in combination with this angle to predict the knee extensor moment contribution. Trunk flexion motion is considered compensation for the posterior shift of the COP position during squatting with restriction of ankle dorsiflexion [7, 12]. However, such an association between the AP-COP position and trunk flexion is not apparent under the natural squatting conditions used in the present study. Neither ankle dorsiflexion nor trunk flexion angle alone appears to explain the AP-COP position during double-leg squatting. Previous studies have shown that an anterior COP position is also associated with the ankle plantar flexor moment [13, 20]. Therefore, the AP-COP position during squatting would be more related to the lower-limb joint kinetics than to the kinematics.

The present study found significant interlimb differences in all interesting variables, including knee extensor moment contribution, ankle dorsiflexion and AP-COP position. However, the LSI of the AP-COP position was the only parameter that could be used for predicting the LSI of the knee extensor moment contribution. A previous study showed that the LSIs of the knee extensor moment were significantly predicted by the LSI of the AP-COP position for patients after anterior cruciate ligament reconstruction [5]. The mean LSI of the knee extensor moment of patients after anterior cruciate ligament reconstruction was 67% [5], which is certainly smaller than that of the healthy population, as shown in the present (94%) and a previous study (87–90%) [13]. The present study found that the LSI of the AP-COP position is still feasible to predict the LSI of knee extensor moment contribution even for the healthy people, despite small asymmetry. Additionally, the LSI of ankle dorsiflexion angle was not significantly associated with the LSI of the knee extensor moment. Although a significant interlimb difference in the ankle dorsiflexion angle was found, the 95% CI of the interlimb difference was 0.4° to 1.6°. Therefore, a small difference in the ankle dorsiflexion would make it difficult to predict the LSI of the knee extensor moment. The LSI of the AP-COP position would be more sensitive than the LSI of the ankle dorsiflexion angle in predicting the LSI in the knee extensor moment contribution. However, as unilateral restriction of ankle dorsiflexion has been reported to lead to interlimb asymmetry in vertical ground reaction force during double-leg squatting [21], the present results may not be applicable to individuals with evident asymmetry in the range of motion of ankle dorsiflexion.

The AP-COP position can predict the knee extensor moment contribution during double-leg squatting independent of the ankle dorsiflexion and trunk flexion angles. These findings suggest the possibility that the knee extensor moment can be coordinated by modifying the AP-COP position without strictly limiting ankle dorsiflexion or increasing trunk flexion, which are concerned about the increased load on the low back. Visual feedback regarding the AP-COP position requires only a force plate and may be useful for controlling knee extensor moment during double-leg squatting exercises. A recent study has shown that a difference in the AP-COP position of approximately 10% of foot length changed the knee extensor moment by 6% without difference in trunk flexion and ankle dorsiflexion [22], which supports the present findings.

The COP position should be guided anteriorly to reduce the knee extensor moment in order not to interfere with graft healing in the early postoperative period after anterior cruciate ligament reconstruction, while the COP position should be guided posteriorly to increase the knee extensor moment in the rehabilitation period to build the strength of the quadriceps. Furthermore, instruction in maintaining a symmetrical AP-COP position using visual feedback regarding the AP-COP position may help to reduce asymmetry of the knee extensor moments after anterior cruciate ligament reconstruction. Patients with knee extensor mechanism problems such as patellar tendinopathy or patellofemoral pain syndrome may also benefit from AP-COP position feedback help them control their knee extensor moment during double-leg squatting.

There are some limitations of this study that should be acknowledged. First, this study examined double-leg squatting without a resistance weight. Therefore, squatting with a resistance weight may result in models that are different from those in the present study. Second, it is unclear whether intervention that focuses on the AP-COP position and ankle dorsiflexion reduces the knee extensor moment without changing trunk flexion. Additional studies should be performed to investigate the effects of instructions focusing on the AP-COP position combined with ankle dorsiflexion on knee extensor moment and hip and trunk biomechanics.

## Conclusion

The present study investigated whether the AP-COP position alone and in combination with ankle dorsiflexion and trunk flexion angles can predict the contribution of knee extensor moment during double-leg squatting. The results showed that AP-COP position was a predictor of the knee extensor moment contribution in univariate analysis and when combined with the ankle dorsiflexion angle and trunk flexion angle. On the other hand, the AP-COP position was not significantly associated with the ankle dorsiflexion or trunk flexion angle. Therefore, intervention focusing on the AP-COP position combined with the ankle dorsiflexion and trunk flexion angles would be useful to coordinate the contribution of the knee extensor moment during double-leg squatting. Furthermore, only the LSI of the AP-COP position was a predictor of the LSI of knee extensor moment contribution. Therefore, the feedback regarding interlimb difference in AP-COP position would be useful to modify the interlimb difference in knee extensor moment contribution.

## Data Availability

The datasets generated and analyzed in the current study are not publicly available due to privacy/ethical reasons but are available from the corresponding author on reasonable request.

## Abbreviations

COP: Center of pressure
AP: Anterior-posterior
LSI: Limb symmetry index
ASIS: Anterior superior iliac spine
PSIS: Posterior superior iliac spine
SD: Standard deviation

## References

[1] Schoenfeld BJ (2010). Squatting kinematics and kinetics and their application to exercise performance. J Strength Cond Res.

[2] Salem GJ, Salinas R, Harding FV (2003). Bilateral kinematic and kinetic analysis of the squat exercise after anterior cruciate ligament reconstruction. Arch Phys Med Rehabil.

[3] Roos PE, Button K, van Deursen RWM (2014). Motor control strategies during double leg squat following anterior cruciate ligament rupture and reconstruction: an observational study. J Neuroeng Rehabil.

[4] Sigward SM, Chan M-SM, Lin PE, Almansouri SY, Pratt KA (2018). Compensatory strategies that reduce knee extensor demand during a bilateral squat change from 3 to 5 months following anterior cruciate ligament reconstruction. J Orthop Sports Phys Ther.

[5] Chan MS, Sigward SM (2020). Center of pressure predicts Intra-limb compensatory patterns that shift demands away from knee extensors during squatting. J Biomech.

[6] Rosen AB, Wellsandt E, Nicola M, Tao MA. Current clinical concepts: clinical management of patellar tendinopathy. J Athl Train. 2021. 10.4085/1062-6050-0049.21. 10.4085/1062-6050-0049.21 PMC9528703 34623447

[7] Kernozek TW, Gheidi N, Zellmer M, Hove J, Heinert BL, Torry MR (2018). Effects of anterior knee displacement during squatting on patellofemoral joint stress. J Sport Rehabil.

[8] Biscarini A, Benvenuti P, Botti F, Mastrandrea F, Zanuso S (2011). Modelling the joint torques and loadings during squatting at the Smith machine. J Sports Sci.

[9] Lorenzetti S, Gülay T, Stoop M, List R, Gerber H, Schellenberg F, Stüssi E (2012). Comparison of the angles and corresponding moments in the knee and hip during restricted and unrestricted squats. J Strength Cond Res.

[10] Fry AC, Smith JC, Schilling BK (2003). Effect of knee position on hip and knee torques during the barbell squat. J Strength Cond Res.

[11] Straub RK, Barrack AJ, Cannon J, Powers CM (2021). Trunk inclination during squatting is a better predictor of the knee-extensor moment than shank inclination. J Sport Rehabil.

[12] List R, Gülay T, Stoop M, Lorenzetti S (2013). Kinematics of the trunk and the lower extremities during restricted and unrestricted squats. J Strength Cond Res.

[13] Flanagan SP, Salem GJ (2007). Bilateral differences in the net joint torques during the squat exercise. J Strength Cond Res.

[14] Kobayashi Y, Kubo J, Matsuo A, Matsubayashi T, Kobayashi K, Ishii N (2010). Bilateral asymmetry in joint torque during squat exercise performed by long jumpers. J Strength Cond Res.

[15] Webster KE, Austin DC, Feller JA, Clark RA, McClelland JA (2015). Symmetry of squatting and the effect of fatigue following anterior cruciate ligament reconstruction. Knee Surg Sports Traumatol Arthrosc.

[16] McClelland JA, Webster KE, Grant C, Feller J (2010). Alternative modelling procedures for pelvic marker occlusion during motion analysis. Gait Posture.

[17] de Leva P (1996). Adjustments to Zatsiorsky-Seluyanov's segment inertia parameters. J Biomech.

[18] Earp JE, Newton RU, Cormie P, Blazevich AJ (2016). Faster movement speed results in greater tendon strain during the loaded squat exercise. Front Physiol.

[19] Song Y, Li L, Albrandt EE, Jensen MA, Dai B (2021). Medial-lateral hip positions predicted kinetic asymmetries during double-leg squats in collegiate athletes following anterior cruciate ligament reconstruction. J Biomech.

[20] Gruben KG, Boehm WL (2012). Mechanical interaction of center of pressure and force direction in the upright human. J Biomech.

[21] Crowe MA, Bampouras TM, Walker-Small K, Howe LP (2020). Restricted unilateral ankle dorsiflexion movement increases interlimb vertical force asymmetries in bilateral bodyweight squatting. J Strength Cond Res.

[22] Ishida T, Samukawa M, Endo D, Kasahara S, Tohyama H (2022). Effects of changing center of pressure position on knee and ankle extensor moments during double-leg squatting. J Sports Sci Med.

